# Flavonoids as Inhibitors of Bacterial Efflux Pumps

**DOI:** 10.3390/molecules26226904

**Published:** 2021-11-16

**Authors:** Martin Waditzer, Franz Bucar

**Affiliations:** University of Graz, Institute of Pharmaceutical Sciences, Department of Pharmacognosy, Beethovenstraße 8, 8010 Graz, Austria; martin.waditzer@gmx.at

**Keywords:** flavonoids, efflux pumps, bacterial membrane, antibiotics, transport proteins

## Abstract

Flavonoids are widely occurring secondary plant constituents, and are abundant in vegetable and fruit diets as well as herbal medicines. Therapeutic treatment options for bacterial infections are limited due to the spread of antimicrobial resistances. Hence, in a number of studies during the last few years, different classes of plant secondary metabolites as resistance-modifying agents have been carried out. In this review, we present the role of flavonoids as inhibitors of bacterial efflux pumps. Active compounds could be identified in the subclasses of chalcones, flavan-3-ols, flavanones, flavones, flavonols, flavonolignans and isoflavones; by far the majority of compounds were aglycones, although some glycosides like kaempferol glycosides with p-coumaroyl acylation showed remarkable results. *Staphylococcus aureus* NorA pump was the focus of many studies, followed by mycobacteria, whereas Gram-negative bacteria are still under-investigated.

## 1. Introduction

### 1.1. Antibiotic Resistance

Antibiotic resistance mechanisms can confer intrinsic, acquired or adaptive resistance. Intrinsic resistance means that all members of a certain bacterial species are naturally resistant to a particular antibiotic or antibiotic substance class. This form of resistance may be mediated by a lack of the antibiotic’s target, reduced cell permeability, chromosomally encoded β-lactamases or target protecting resistance mechanisms. Intrinsic resistance mechanisms may be constitutively expressed or induced by the respective antibiotic [1,2,3]. However, bacteria can also acquire resistance due to mutations or horizontal gene transfer [4]. In contrast to intrinsic and acquired resistance, adaptive resistance is a transient and not inheritable tolerance to antibiotic insults. This is a result of altered gene expression, which leads to slow growth or upregulation of antibiotic resistance determinants like efflux pumps or target protecting proteins. Antimicrobials are usually active against growing cells and this is why restriction of growth may be a resistance determinant itself [2,5].

### 1.2. Bacterial Efflux Pumps

Efflux pumps (EP) are energy driven transporters and belong to the huge number of transport proteins (which are membrane proteins) responsible for the transport of molecules into and out of cells. EP can be classified as electrochemical potential driven transporters (secondary active transporters) or primary active transporters. Primary active transporters transport their substrates in or out of cells against a concentration gradient by using the energy released by ATP hydrolysis. Secondary active transporters use the concentration gradient generated by a primary active transport mechanism to drive the transport of their substrates against a concentration gradient. The generated concentration gradient may be a H^+^ or Na^+^ gradient which is the driving force for the secondary active transport. The first report of drug efflux activity in bacteria was in 1980 when tetracycline efflux was reported in *Escherichia coli* isolates [5,6]. Some drug efflux pumps can specifically extrude a certain drug, but the majority of drug efflux pumps are multidrug efflux pumps which lead to the efflux of many chemically similar and unrelated substrates. Members of antibiotic multidrug efflux pumps in bacteria belong to 6 families: The ATP-binding cassette (ABC) superfamily, the major facilitator superfamily (MFS), the resistance-nodulation-cell-division (RND) superfamily, the drug/metabolite transporter (DMT) superfamily, the multidrug/oligosaccharidyl-lipid/polysaccharide (MOP) flippase superfamily, and the proteobacterial and antimicrobial compound efflux (PACE) family, which harbors efflux systems common in proteobacteria and which is the newest bacterial multidrug efflux pump family discovered in 2015 [6,7,8,9,10]. A recent review by Du et al. not only illustrates representative structures of multidrug transporters and tripartite assemblies but also discusses mechanistic function and regulation thereof [11].

The overexpression of bacterial multidrug efflux pumps due to mutations or adaptive responses usually confers low-level resistance to antibiotic pump substrates. This decreases the intracellular concentration of antibiotics and facilitates bacterial survival in environments that contain suboptimal, low antibiotic concentrations which may be the result of a wrong dosing regimen, bad compliance or limited diffusion of the antibiotic in vivo. The low-level resistance gives bacteria time to adapt to the antibiotic challenge by initiating time consuming adaptive responses like the inducible expression of antibiotic degrading enzymes or the even more time-consuming biofilm formation. The most relevant and most reported consequence of the efflux pump mediated gain in time is the increased probability of the emergence of mutations that confer high-level resistance to the efflux pump substrates. Furthermore, low-level resistance conferring EP have been reported to be essentially required to act in concert with other resistance mechanisms in order to create clinically relevant high-level resistance to certain antibiotics. Several EP, including for example the tetracycline efflux pumps, are sufficient per se to confer high-level resistance to certain antibiotics, and most of them are located on mobile genetic elements [5,6,7,12,13,14,15,16,17].

MFS efflux pumps are the most relevant efflux determinants in antibiotic resistance of Gram-positive bacteria. In *Staphylococcus aureus*, the fluoroquinolone extruding MFS pump NorA might contribute to fluoroquinolone resistance by acting in concert with topoisomerase target mutations and several norfloxacin resistant clinical isolates have been reported to overexpress NorA [5,17,18,19]. Enterococci are the most important nosocomial pathogens after *S. aureus* and it has been shown that *Enterococcus* isolates harbor a plasmid which encodes MefA, the ABC efflux pump MsrA/B and a β-lactamase. This plasmid confers high level resistance to erythromycin and ampicillin [19]. In *Mycobacterium smegmatis*, the MFS transporter LfrA represents a prominent EP and confers low-level resistance to fluoroquinolones, but also other toxic compounds such as EtBr [20].

RND pumps are the most relevant efflux determinants in the antibiotic resistance of Gram-negative bacteria. Efflux pumps of Gram-negative bacteria can contribute to the development and maintenance of fluoroquinolone resistance and the overexpression of efflux pumps in fluoroquinolone resistant Gram-negative bacteria has been observed frequently [5,6,7,19,21,22]. Many studies have confirmed the important contribution of the overexpression of the RND efflux pump AcrAB-TolC to high-level fluoroquinolone resistance of isolates of *E. coli* [23]. *Salmonella* species may be food borne pathogens and their AcrAB-TolC pump homolog has also been suggested to be essential for high-level fluoroquinolone resistance by acting in concert with target mutations. The inactivation of the pump gene *acrB* in high-level fluoroquinolone resistant *Salmonella* isolates led to a 32-fold reduction in the MIC, and this made these bacteria susceptible to fluoroquinolones as this MIC was above the clinical breakpoint concentration [6,19]. The RND efflux pump CmeABC of the human enteropathogenic *Campylobacter jejuni* is an example of a multidrug efflux pump that is involved in the stepwise development of macrolide resistance. In vitro exposure of *C. jejuni* to large concentrations of erythromycin first led to the emergence of low-level resistance caused by target mutations in ribosomal proteins. This scenario was followed by mutation driven CmeABC overproduction, which led to intermediate-level resistance. Finally, mutations in ribosomal RNA (23S rRNA) occurred and this led to high-level erythromycin resistance [6].

### 1.3. Mechanisms of Efflux Pump Inhibition

**Figure 1:**
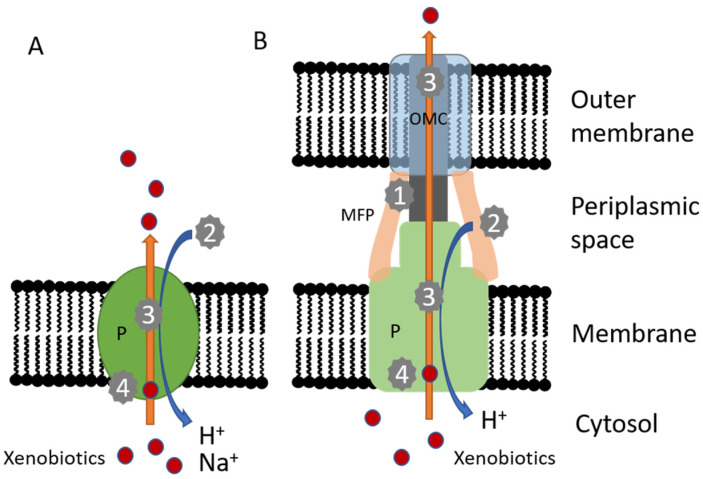
Scheme of secondary active multidrug efflux pumps with selected possible targets of inhibitors. (**A**) Single component pump (e.g., MFS pump), (**B**) Tripartite RND pump. Both types are driven by an electrochemical potential. Inhibitors might (1) interfere with the assembly of the tripartite pump, (2) inhibit the electrochemical gradient, (3) block the efflux pump channel or outer membrane protein exit channel or (4) interfere either with the binding site of efflux pump substrates or inhibit the essential conformational changes during the efflux process. P pump, MFP membrane fusion protein, OMC outer membrane channel.

Several mechanisms of efflux pump inhibition have been reported [6,24,25,26,27,28,29,30]. Hence, conclusions on direct inhibition of efflux pumps based on findings of increased accumulation or decreased efflux of EP substrates or increased sensitivity of bacteria to compounds known to be EP substrates have to be done with great caution. A point-by-point list of possible mechanisms is presented below (see also Figure 1):

- Inhibition or down-regulation of EP expression by targeting local transcriptional regulators, global transcriptional regulators, other components of the regulatory system or posttranscriptional steps of gene expression;
- blocking of the assembly of protein components of tripartite efflux pumps by targeting protein-protein interfaces;
- inhibition of the pump´s energy source by the decrease of the ATP supply or the inhibition of the proton motive force;
- inhibition of phosphorylation steps that induce or enhance the activity of regulatory proteins or efflux pump proteins;
- application of antibodies against pump proteins or other protein components of tripartite efflux pumps;
- blocking of the efflux pump channel or the outer membrane protein exit channel;
- application of a small mimic/decoy molecule that is recognized and effluxed in preference to the antibiotic;
- application of small efflux pump inhibitory molecules that competitively block the efflux pump drug-binding site and that exert steric hindrance of substrate access to the binding site, as well as
- application of small efflux pump inhibitory molecules that non-competitively inhibit the essential conformational changes of efflux pumps during the efflux process.

### 1.4. Flavonoids

Plants and their extracts contain a huge number of compounds with multifaceted chemical structures and plants can be regarded as potent source of efflux pump inhibitors (EPIs). They have always been in competition with phytopathogenic bacteria and in the course of evolution bacteria developed defense mechanisms like efflux pumps in order to protect themselves from antibacterial substances secreted by microbial competitors and plants. In the course of subsequent evolutionary events, plants gained the ability to synthesize EPIs in order to enhance the effect of their secreted antibacterial compounds. Plant antibacterials may be weak antibiotic compounds which only exert a sufficient antibiotic effect in combination with an EPI. Such synergistic effects have often been observed to be the cornerstone of diverse pharmacological effects of plant extracts and this synergism of plant extracts can be easily exemplified by the enhanced effect of an antibacterial compound in combination with an EPI [31,32,33,34,35,36,37,38,39,40].

Flavonoids are herbal secondary metabolites and more than 8000 flavonoid-like structures have been reported. Chemically, they are 2-phenyl-benzo-γ-pyrones and the modification of this core structure leads to the impressive high number of naturally occurring flavonoids. The oxidation pattern of the C-ring is the criteria for dividing them into distinct subclasses like flavanes, flavanols and flavanones (all with a reduced C-2,3-bond), flavones, flavonols and anthocyanidins. In isoflavonoids, the B-ring is attached to position 3 of ring C, whereas chalcones are α,β-unsaturated ketone derivatives which can be easily cyclized to flavanones and which are the biosynthetic precursors of the other mentioned flavonoid subclasses [41]. Major structural diversity of flavonoids results from hydroxylation, methoxylation and glycosylation of the basic structures [42]. These substances protect plants from fungal infections and UV radiation, provide color to flowers, and they contribute to photosensitization, energy transfer, respiration and photosynthesis control, morphogenesis, sex-determination and energy transfer. The dietary intake of flavonoids is about 1–2 g/day and a plethora of biological activities has been reported for flavonoids. Examples of their reported effects are anti-inflammatory, antibacterial, antiviral, antiallergic, cytotoxic, antitumor or antioxidant activities [43,44,45]. Resistance modulating effects due to efflux pump inhibition by naturally occurring flavonoids of different subclasses will be presented in detail in the following section.

## 2. Methods for Measuring Efflux across Bacterial Cell Walls in Bacteria

The efflux pump inhibitory effect of a compound is usually determined by drug susceptibility tests followed by the direct measurement of efflux or accumulation of preferably fluorescent pump substrates in the presence and absence of the examined sample [29,30,46,47,48,49]. Drug susceptibility tests are resistance modulation assays used to determine the enhancement of the effect of certain antibiotics and the concomitant decrease of the minimal inhibitory concentrations (MICs) of these antibiotics against certain bacteria exerted by sub-inhibitory concentrations of the examined sample. The intensity of the potentiation of the antimicrobial effect may often be described by a modulation factor (MF), describing the x-fold decrease of the antibiotic MIC in presence of the modulator compound [50]. In order to assure that the observed antibiotic potentiation as well as a subsequently determined increase of the intracellular accumulation of a fluorescent substrate is due to efflux pump inhibition, strains with deleted or overexpressed efflux pumps have to be used as reference strains [51,52,53]. No effect in efflux pump deleted mutants and an enhanced effect in efflux pump overexpressing mutants then might rule out other mechanisms like cell membrane permeabilization. Fluorescent dyes like EtBr, Nile red or 1,2′-dinaphthylamine are usually used as fluorescent substrates of EP in efflux assays [46]. EtBr is a substrate of many EP like the RND pump AcrAB-TolC of Enterobacteriaceae and it shows fluorescence only when it is in the cell interior in nonpolar and hydrophobic environments, while the lipophilic dyes Nile red and 1,2′-dinaphthylamine bind to membrane phospholipids in the periplasmic space and these bound dyes fluoresce more strongly than unbound dyes in aqueous solution [46].

In principle, either the accumulation or the efflux of a fluorescent substrate is evaluated in real time using a microplate fluorimeter [50,54] or, alternatively, a real-time PCR instrument [55]. Recently, detailed protocols for analyzing influx and efflux of fluorescing substrates in mycobacteria have been published by Rodrigues et al. [56]. Using this type of assays more extensively, it becomes obvious that not only the efflux inhibitory activity of compounds can be measured, but also valuable information on cell wall and membrane permeability can be obtained. As a consequence, these assays do not directly answer the question of whether a specific compound can be designated as an efflux pump inhibitor. Increased accumulation of a fluorescent substrate might also result from an increased permeability of the bacterial cell wall or membrane, hence additional experiments have to be undertaken in order to evaluate the effect of a potential EPI on membrane/cell wall permeabilization [57]. Frequently, this is done by measuring emission/quenching of pairs of fluorescence dyes with different permeation properties, e.g., SYTO9/propidium iodide [58].

Furthermore, mass spectrometry has been applied to measure the accumulation of a substrate by measuring the substrates´ depletion from a spent liquid medium [59]. This approach will be useful if non-fluorescent efflux pump substrates are used. It is also useful for the determination of efflux pump inhibitory activities of complex mixtures which might contain molecules that cause optical interference during fluorescence-based assays. However, direct interaction of EP substrates and compounds from plant extracts or isolated compounds cannot be excluded in the MS based assays as well.

## 3. Flavonoids as Inhibitors of Multidrug Bacterial Efflux Pumps

**Figure 2:**
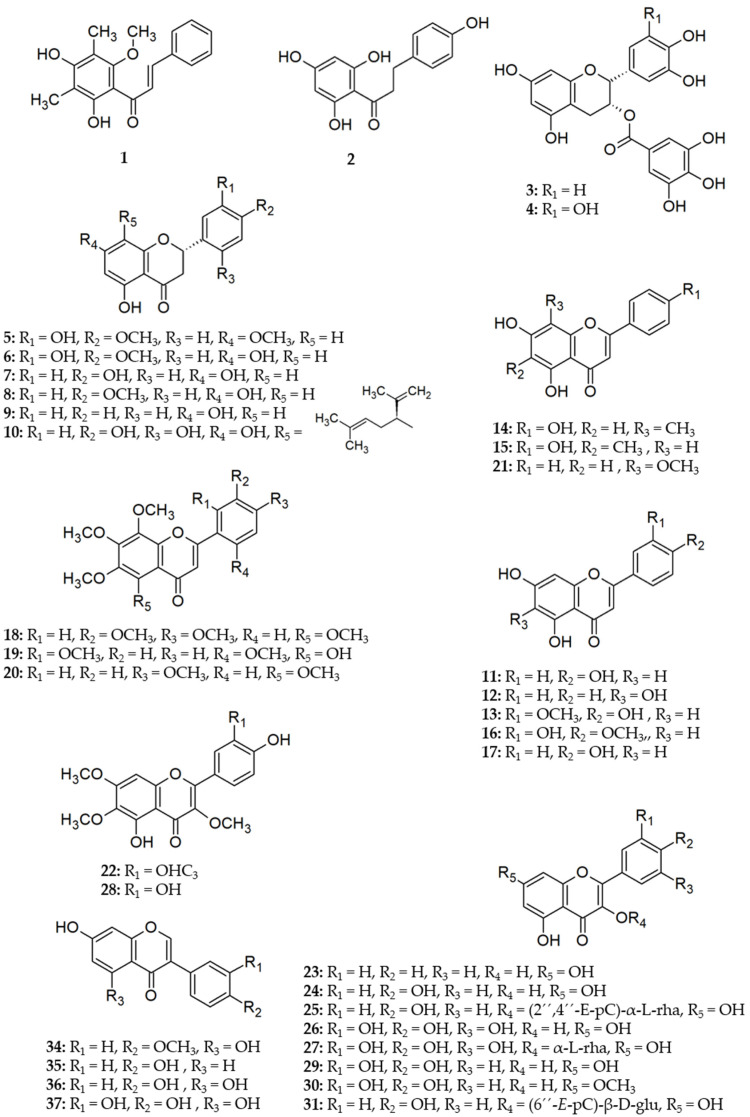
Structures of naturally occurring flavonoids reported as efflux pump inhibitors. Compounds of chalcone, flavan-3-ol, flavanone, flavone, flavonol and isoflavone subclass are shown here, for flavonolignans see Figure 3. *E*-pC = *E*-p-coumaroyl; α-l-rha = α-l-rhamnoside; β-d-glu = β-d-glucoside.

**Figure 3:**
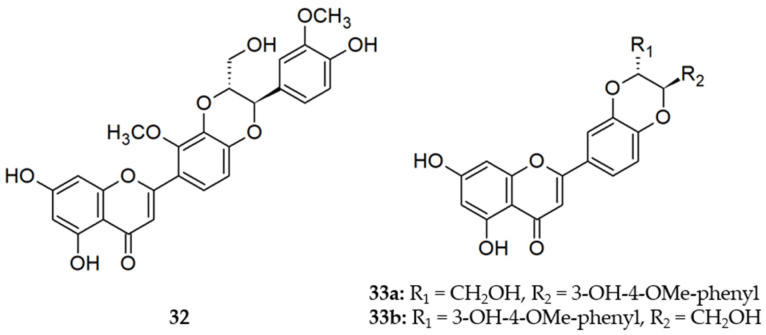
Structures of flavonolignans **32** and **33** (i.e., diastereomeric mixture of **33a** and **33b**).

Compounds being active as bacterial efflux pump inhibitors seem to be common in land plants [60]. Considering EPIs of plant flavonoids, the main focus has been on the efflux inhibition in Gram-positive bacteria. Flavonoids have been investigated most frequently for EPI activity on the NorA pump in studies with defined efflux pump targets. In second place, concerning the frequency of reports, investigations on non-tuberculous mycobacteria follow, whereas only scarce data exist on Gram-negative bacteria. This might be a consequence of the higher intrinsic resistance of Gram-negative bacteria against antibiotics and other exogenous compounds as well as a consequence of the complexity of the substrate recognition of RND transporters. A detailed overview of flavonoids for which inhibition of efflux has been reported based on results from efflux or accumulations assays or based on comparison of strains with different expression levels of MDR pumps is presented in Table 1; structures of these flavonoids are depicted in Figure 2 and Figure 3.

This review was prepared by searching literature in PubMed (https://pubmed.ncbi.nlm.nih.gov/; accessed on 8 November 2021) and SciFinder (https://scifinder.cas.org/; accessed on 8 November 2021) for the search terms flavonoid(s) in combination with efflux, efflux pump(s), bacteria, bacterium, microbial, P-gp, NorA. For flavonoids/efflux, only 218 reports could be found, of which 31 publications remained for further evaluation after refining for bacteria.

So far, naturally occurring chalcones have been infrequently reported to inhibit bacterial efflux pumps. From *Dalea versicolor* (Fabaceae), among other flavonoids, 4′,6′-dihydroxy-3′,5′-dimethyl-2′-methoxychalcone (**1**) has been isolated. When tested against *S. aureus* wild type and a corresponding *norA* knockout mutant, **1** at 10 mg/L increased the sensitivity of both strains to berberin, erythromycin and tetracyclin. As the effect was lower in the case of the knockout mutant, the authors concluded that the mode of resistance modifying action was associated with the NorA pump [61]. For comparison, the *trans*-chalcone of synthetic origin seems to be less active, since it was found to increase at 125 mg/L accumulation of EtBr in *Bacillus subtilis* with a comparable potency as chlorpromazine (CPZ); however, it failed to show activity in *E. coli*, *M. smegmatis*; *M. aurum* and *M. bovis* BCG, respectively [78]. The dihydrochalcone **2** has been regarded as an inhibitor of the NorA pump in the overexpressing strain *S. aureus* 1199B due to reduction of MIC of the NorA substrates norfloxacin and EtBr (at 64 mg/L 4- and 2-fold reduction of MIC, respectively) [62]. However, no further EtBr accumulation/efflux experiments have been performed to substantiate this assumption. In the same study, the flavanones hesperetin (**6**), naringenin (**7**) and the flavonol glycoside myricitrin (**26**) revealed potential NorA inhibition by reduction of MIC values of norfloxacin and EtBr at 64 mg/L [62].

EPI activity has been detected for catechins. Epicatechin gallate (**3**) and epigallocatechin gallate (**4**) proved to be weak inhibitors of EtBr efflux in *S. aureus* 1199B, a NorA overexpressing strain, but could reduce norfloxacin MIC fourfold. Interestingly, the additional OH group in **4** led to increased activity [53]. However, flavonoids may also stimulate the efflux pump mediated transport of certain substrates. Epicatechin gallate (**3**) stimulated the activity of the *S. aureus* MFS pump NorA at concentrations less than or equal to 20 µM, while higher concentrations mediated efflux pump inhibition. However, IC_50_ proved to be higher than 100 µM for **3** and **4**, respectively [53]. A study of compound **4** on 17 macrolide resistant and three reference strains of the Gram-negative *C. jejuni*, a major cause of food-borne gastrointestinal infections, revealed resistance modifying effects to ciprofloxacin, tetracyclin and macrolides, which could partly be related to CmeABC and CmeDEF pumps by comparing antibiotic sensitivities of parental and efflux pump gene knockout strains [52,63]. A 4—64-fold decrease of the MICs to azithromycin, clarithromycin and dirithromycin was exerted by combination with **4** at 0.25 × MIC in 60% of the tested isolates.

A study conducted by [64] showed that sub-inhibitory concentrations (0.25 × MIC) of the flavanones eriodictyol-7,4′-dimethyl ether (**5**) and naringenin-4′-methyl ether (**8**) isolated from *Chromolaena odorata* decreased the MIC of the universal efflux pump substrate EtBr against MRSA clinical isolates and the reference MRSA strain ATCC 33591 by 4- or 8-fold. Therefore, these compounds exceeded the enhancing effect of a concentration of 50 mg/L reserpine, which is a common inhibitor of *S. aureus* efflux pumps. The chalcone 2′,4-dihydroxy-4′,5′,6′-trimethoxychalcone was only active against the clinical isolates, and therefore not included in Table 1. Mutually tested flavonols like methyl ethers of quercetin and kaempferol showed no efflux pump inhibitory activity. The MIC of EtBr against a methicillin-sensitive *S. aureus* strain was not changed or was just changed twofold in combination with the two effective flavanones [64]. Another flavanone, pinocembrin (**9**), isolated from *Alpinia katsumadai* seeds, showed increased EtBr accumulation at 64 mg/L, comparable to CPZ in *M. smegmatis*, but was less effective than CPZ in the EtBr efflux assay [50]. The same compound was also obtained from *Alpinia calcarata*, but could not prove its EtBr efflux inhibitory effects in *S. aureus* 1199B when compared to the corresponding wild type strain *S. aureus* 1199 and the NorA knockout mutant K1758 [65]. Sophoraflavanone G (**10**) showed a synergistic effect when combined with norfloxacin against the NorA overexpressing *S. aureus* 1199B (16-fold reduction in the MIC of norfloxacin), which could be confirmed by an EtBr efflux experiment. Even more interestingly, this increased norfloxacin sensitivity could be confirmed in vivo [66], supporting the concept of EPI to be applied in combination with known antibiotics in infectious diseases.

Most frequently, flavones have been reported as inhibitors of bacterial efflux pumps. Apigenin (**11**) proved to be less active as an EtBr accumulation inhibitor compared to the corresponding flavonol kaempferol (**24**) [59]. Baicalein (**12**) showed inhibition of TetK efflux pump based on results in MRSA strains having the *tetK* gene as well as by studying tetracycline transport in everted membrane vesicles from *E. coli* KAM32/pTZ1252 cells that expressed TetK derived from *S. aureus* and from *E. coli* KAM32 cells [67]. In a study of Solnier et al. on mycobacteria [68], more lipophilic structures like the methoxylated flavones nobiletin (**18**), skullcapflavone II (**19**), tangeretin (**20**) and wogonin (**21**) as well as flavones lacking substituents at the C-2 aryl ring (baicalein (**12**), wogonin (**21**)) which might have a higher affinity for the lipid-rich mycobacterial cell envelope were selected. At half of their MICs, all tested compounds **12** (16 mg/L), **18–21** (64 mg/L) improved the accumulation of EtBr in *M.aurum* compared to the negative control. Nobiletin (**18**) achieved the highest EtBr-accumulation level in *M. aurum* equal to verapamil, whereas compounds **12** and **18**, lacking C-2 aryl ring substituents, were the least effective against *M. aurum*. In *M. smegmatis*, an increase in EtBr accumulation to a similar extent as CPZ was caused by baicalein (**12**) at 16 mg/L and nobiletin (**18**) at 32 mg/L. All other compounds (**19–21**) were more active than the negative control but did not reach the levels of the standard EPIs verapamil and CPZ. Interestingly, the additional 3′-methoxy group in **18** compared to **20** significantly improved the efflux inhibitory activity. The authors could not find a correlation of strong modulatory effects when sub-inhibitory concentrations of the flavones were tested in combination with rifampicin or EtBr in *M. smegmatis* or *M. aurum* and putative efflux activity of the compounds. This might be due to the different exposure times of the bacteria to the flavones and EtBr in accumulation and modulation assays (1 hr. vs. 72 hrs., respectively). The same discrepancy between synergistic activity with antibiotics and missing efflux activity in an EtBr efflux assay was observed for diosmetin (**16**). Although significantly decreasing MIC values for norfloxacin, ciprofloxacin and streptomycin in NorA overexpressing *S. aureus* 1199B as well as EMRSA-15, no inhibition of EtBr efflux in *S. aureus* 1199B could be confirmed [71]. In a study by Guz et al. [72] compound **16** indicated inhibition of NorA efflux of berberin, based on the assumption that increasing sensitivity of *S. aureus* to berberin is mainly due to the inhibition of berberin efflux by the NorA efflux pump [37].

In a synergy-directed fractionation process based on the checkerboard assay and LC-MS analysis, three flavonoids from the leaves of *Hydrastis canadensis* could be isolated. Among them, 6-desmethyl sideroxylin (**14**) and 8-desmethyl sideroxylin (**15**) proved to be synergistically active with antibacterial berberin and inhibited EtBr efflux, whereas no EPI activity could be seen in the *norA* knockout mutant. The parent compound sideroxylin was much less potent [70].

In the aerial parts of the TCM plant *Artemisia rupestris*, the flavone chrysoeriol (**13**) as well as the flavonol aglycones chrysosplenetin (**22**) and penduletin (**28**) were detected as efflux inhibitors in *S. aureus* strains, including the MRSA strains EMRSA-15 and EMRSA-16, which are highly problematic in clinical settings in the UK [69]. In this recent study, more in depth investigations on the mode of efflux inhibition were performed, revealing a significantly decreased expression of NorA at the mRNA level caused by compounds **13**, **22** and **28**. In-silico docking studies with the NorA protein confirmed interactions of different types (hydrogen bond, van der Waals, carbon-hydrogen bond and pi-alkyl interactions). In addition, for chrysoeriol (**13**) resistance, modulatory effects in both EMRSA strains were supported by finding in-silico-interactions with the penicillin binding protein PBP2a [69].

Among flavonol aglycones, kaempferol (**24**) and rhamnetin (**30**) proved to the most active in a mass spectrometry supported assay measuring extracellular EtBr levels, avoiding the possible fluorescence quenching effects of flavonoids [59]. Galangin (**23**) and kaempferol (**24**) exerted much higher reductions of EtBr MIC values in the NorA overexpressing strain *S. aureus* 1199B compared to the corresponding wild type strain *S. aureus* 1199, and were devoid of activity in the NorA knockout mutant K1758, indicating the NorA pump as the target. Interestingly, no activity could be seen in galangin-3-methyl ether [65]. In silico docking to AcrB revealed the efflux pump inhibitory activity of quercetin (**29**) and the interaction has been shown to be stabilized by hydrogen bonds and hydrophobic interactions between phenylalanine side chains and aromatic rings of the flavonoid. This might occur in the lower part of the distal binding pocket [74,77]. In the study by [74], **29** exerted at 200 µM inhibition of Nile red efflux from *E. coli* BW25113 wild type strain to a level which was in the range of the *acrB* deletion mutant, but it failed to synergize with tested antibiotics. In addition, the authors could show that the test compound **29** at 100 mg/L did not permeabilize the outer membrane.

Whereas most of the studies on flavonols revealed the efflux pump inhibitory effects of flavonol aglycones (**22, 24, 26, 28, 29, 30**), only a few reports have been published on flavonol 3-*O*-glycosides. Holler et al. [73] reported the NorA inhibitory activity of kaempferol-3-*O*-α-l-(2,4-bis-*E*-p-coumaroyl)rhamnoside (**25**) isolated from *Persea linguae*. This compound is one of the most potent EPIs from natural sources discovered so far. *Persea linguae* is used as a traditional medicinal plant by the Chilean Huilliche people. The sub-inhibitory concentration of 1.56 mg/L of this kaempferol rhamnoside enhanced the effect of ciprofloxacin against the NorA overexpressing strain *S. aureus* 1199B and the same degree of synergy was only achieved using a reserpine concentration of 6.25 mg/L. No ciprofloxacin enhancing effect was observed in the *norA* knockout strain. Furthermore, the kaempferol rhamnoside inhibited the EtBr efflux from NorA overexpressed *S. aureus* 1199B with an IC_50_ value of 2 µM, whereas the IC_50_ of reserpine was determined to be 9 µM. This extent of EtBr efflux inhibition is among the highest found in this MDR system. The putative NorA inhibition of **25** was confirmed by constructing everted membrane vesicles enriched with NorA and measuring Hoechst 33342 efflux.

In case of a compromised activity in vivo due to deglycosylation, there might still be the option of topical application against skin infections, particularly those caused by community associated MRSA. The 2,3-p-coumaroyl isomer of this rhamnosyl glycoside showed no acute toxicity in mice at 20 mg/kg/day [73]. In addition, myricitrin (myricetin-3-O-α-L-rhamnoside) (**27**) [62] and tiliroside (kaempferol-3-*O*-β-d-(6″-*E*-p-coumaroyl) glucopyranoside) (**31**) from *Herissantia tiubae* have been reported to inhibit NorA [75]. In the case of **31**, this was concluded from norfloxacin (8-fold reduction of MIC) and EtBr (64-fold reduction) resistance modulating activity in the NorA overexpressing strain *S. aureus* 1199B when tested at 32 mg/L, at 64 mg/L MIC of norfloxacin was reduced 16-fold, indicating dose dependency [75]. Comparison of the activity of **31** and **25** points towards the importance of increased lipophilicity in the case of the dicoumaroylated rhamnoside **25**.

In the earliest studies on flavonoid EPIs, the flavolignan 5′-methoxyhydnocarpin D (5′-MHCD) **32**, biosynthesized by various *Berberis* species, was found to be an inhibitor of NorA. As a result, 5′-MHCD enhanced the effect of the relatively weak antimicrobial alkaloid berberine [37,76]. It should be mentioned that synthesized derivatives of 5′-MHCD inhibited growth of the *S. aureus* RN4222 wild type strain at sub-inhibitory concentrations of berberin (30 mg/L) at lower concentrations than 5′-MHCD (Guz et al. 2001). Silybin (**33**) was obtained as a diastereomeric mixture **33a** and **33b** by crystallization from silymarin, the hepatoprotective flavonolignan mixture from the fruits of *Silybum marianum*, and was assayed equally to **32**, but was not as active [76]. Structures of compounds **32** and **33a,b** are presented in Figure 3.

The significant efflux inhibitory activity of biochanin A (**34**) in *M. smegmatis* [49] inspired further studies for synthesis of 3-phenylquinolones, which proved to be potent efflux inhibitors in *Mycobacterium avium* [79]. The significant efflux inhibitory effects of biochanin A (**34**) is also supported by the study of Morel et al. [51]. The Fabaceae *Lupinus argenteus* served as a rich source of isoflavones. Using an *S. aureus* wild type strain (8325-4) and a *norA* knockout mutant (KLE-8), by comparing the resistance modulatory effects of biochanin A (**34**), genistein (**36**) and orobol (**37**) at 10 mg/L in combination with berberin, compound **34** proved to be the most potent. In this case, resistance modulatory effects were in line with results of a berberine accumulation assay in *S. aureus*, showing a higher activity of **34**, followed by **37** and **36** [51]. The soy isoflavone daidzein (**35**) has been reported to inhibit efflux pumps of Gram-negative bacteria. In the course of an in-silico screening, **35** has been shown to interact with the distal binding pocket of the RND pumps AcrB of *E. coli* and MexB of *P. aeruginosa*. Confirmation of EPI activity was done in EtBr accumulation assays, where **35** showed increased rates of EtBr fluorescence of 16.36% and 20.37% in *E. coli* and *P. aeruginosa*, respectively [77].

The efflux pump modulatory effects and the corresponding mechanisms of flavonoids are versatile and different among specific flavonoids and bacterial species. According to the previously conducted research on flavonoid mediated inhibition of bacterial efflux pumps, an average SAR cannot be drawn on a sound basis. Apart from the general problem of reproducibility, this may be due to the fluorescence quenching phenomenon, the application of different assays or the use of different efflux pump substrates. To answer the question of mechanism of action of flavonoids as bacterial EPIs, only a few attempts have been made, as whole-cell phenotypic assays have been applied almost exclusively. Lan et al. [69] showed that compounds **13**, **22** and **28** were able to reduce NorA expression at the mRNA level, but also showed in-silico interactions with the predicted binding site of NorA. Similarly, for daidzein (**35**), interaction with the distal binding pocket of the RND pump AcrB of *E. coli* and MexB of *P. aeruginosa* could be seen [77].

Quantification of efflux inhibitory activity mostly is done by referring to reference EPIs like verapamil, reserpine, CPZ or CCCP, which allows comparison of potencies of individual compounds within an experimental setup. In a few cases, quantification has been attempted by measuring the relative fluorescence or normalized fluorescence data within the last 10 min of the assay [50], or by establishing dose response curves [49,59].

## 4. Inhibition of Eukaryotic Multidrug Transporters by Flavonoids

When evaluating the potential application of flavonoids as EPIs in combination with antibiotics to treat infectious diseases, an important issue is their selectivity for bacterial efflux pumps. In a recently published review by Chambers et al. [80], the role of flavonoids in eukaryotic and bacterial MDR has been discussed, revealing a significant overlap of eukaryotic and bacterial MDR modulating effects of flavonoids. Fang et al. [81] investigated the P-gp inhibitory effects of 39 flavonoids on rhodamin-123 and daunomycin uptake models in KB cells and P-gp overexpressing KB cells. Some structural features which enhanced the uptake of substrates could be identified, indicating P-gp inhibition. Favorable structural elements which could reverse MDR were designated as 5-OH, 5-OCH_3_, 6-OH, 7-OCH_3_, 3′-OH, and 4′-OH groups. Among others, baicalein (**12**), tangeretin (**20**), kaempferol (**24**), and quercetin (**29**) proved to be effective inhibitors of P-gp [81]. Ferreira et al. reviewed flavonoids as agents reversing P-gp mediated drug resistance [43] in detail. Docking studies of a flavonoid database with full-length three-dimensional structure of the human P-gp revealed at least two prenylated flavonoids (3′-*O*-methyl-5′-methoxy-diplacone and tomentodiplacone N, respectively) whose MDR reversal activities were confirmed in vitro [82]. The main flavonoid metabolites consisting of glucuronides and sulphate conjugates are more likely to interact with multidrug resistance associated proteins (MRPs). It has been observed that phase II metabolites of quercetin (**29**) are equally potent or even better inhibitors of MRP1 and MRP2 than the parent compound quercetin itself [43]. Having these tools at hand, flavonoids identified in the present review as potential EPIs in bacteria could be checked for P-gp binding properties in future studies for establishing data on selectivity.

## 5. Conclusions

The assays applied for detection of efflux pump inhibition in bacteria mainly follow changes in intra- or extracellular EP substrate levels. In addition, the comparison of resistance modifying effects of flavonoids in parental strains and their knockout mutants or EP overexpressing strains provide valuable information concerning the inhibition of bacterial efflux pumps. Potential alterations of membrane permeability by flavonoids have only been shown in a few studies, and should be included in future experimental setups in order to get sound conclusions on direct EP—flavonoid interactions. However, direct binding of flavonoids to specific EP substructures has only been shown in exceptional cases, such as for RND pumps of Gram-negative bacteria or NorA in *S. aureus*. In the course of this review, numerous reports on flavonoids as bacterial efflux pumps have been summarized, the flavone subclass being the richest one in terms of numbers. Promising structures have also been found among flavanones, flavonol glycosides, flavonolignans and isoflavones.

Future studies on plant derived EPIs should consider several issues. One criterium would be selectivity for bacterial vs. eukaryotic transporters. Furthermore, intracellular availability of potential EPIs in infected cells should be evaluated, including their cytotoxicity in mammalian cells. In addition, phenotypic whole-cell assays for EP inhibition should be accompanied by assays for membrane permeabilization as well as in-silico studies on potential EP binding sites. Finally, in order to take the next step towards clinical application of antibiotic—EPI combinations, the frequently reported resistance modulating effects of a number of flavonoids, even at low µM levels, should be confirmed by in vivo models.

## Figures and Tables

**Table 1:** Naturally occurring flavonoids with reported efflux inhibiting effects in bacteria.

Flavonoid Subclass/Compound	Plant Source	Bacterial Strains	Efflux Pump Affected	Activity	Reference
*Chalcones/Dihydrochalcones*					
4′,6′-Dihydroxy-3′,5′-dimethyl-2′-methoxychalcone (**1**)	*Dalea versicolor*	*S. aureus* NCTC 8325-4, K1712, K1748	NorA	Increasing sensitivity to antibiotics, smaller effect in NorA knockout strain	[61]
Phloretin (**2**)	n.a.	*S. aureus* 1199B	NorA	Decrease of nor-floxacin and EtBr MIC	[62]
*Flavan-3-ols*					
Epicatechin gallate (**3**)	n.a.	*S. aureus* 1199B, XU-212, RN4220, ATCC 25923	NorA, TetK, MsrA	Stimulating efflux at concentrations less than or equal to 20 µM; inhibition at higher concentrations	[53]
Epigallocatechin gallate (**4**)	n.a.	*Campylobacter jejuni* macrolide resistant strains, reference strains and pump knockout mutants	CmeABC, CmeDEF	EPI against sensitive and resistant *Campylobacter* isolates	[52,63]
	n.a.	*S. aureus* 1199B, XU-212, RN4220, ATCC 25923	NorA, TetK, MsrA	Weak inhibition of EtBr efflux	[53]
*Flavanones*					
Eriodictyol-7,4′-dimethyl ether (**5**)	*Chromolaena odorata*	*S. aureus* ATCC 33591 (MRSA); clinical MRSA isolates	n.s.	Decrease of EtBr MIC; only weak activity when tested on MSSA	[64]
Hesperetin (**6**)	n.a.	*S. aureus* 1199B	NorA	Decrease of nor-floxacin and EtBr MIC	[62]
Naringenin (**7**)	n.a.	*S. aureus* 1199B	NorA	Decrease of nor-floxacin and EtBr MIC	[62]
Naringenin-4′-methyl ether (**8**)	*Chromolaena odorata*	*S. aureus* ATCC 33591 (MRSA); clinical MRSA isolates	n.s.	Decrease of EtBr MIC; only weak activity when tested on MSSA	[64]
Pinocembrin (**9**)	*Alpinia katsumadai*	*Mycobacterium smegmatis* mc^2^ 155	n.s.	Stronger inhibition of EtBr efflux than EtBr accumulation	[50]
	*Alpinia calcarata*	*S. aureus* 1199, 1199B, K1758	NorA	No indication for EPI activity	[65]
Sophoraflavanone G (**10**)	*Sophora alopecuroides*	*S. aureus* 1199B	NorA	Synergistic effect when combined with norfloxacin against *S. aureus* 1199B; synergistic effect with norfloxacin in vivo; EtBr efflux inhibition	[66]
*Flavones*					
Apigenin (**11**)	n.a.	*S. aureus* NCTC 8325-4	n.s.	Dose dependent inhibition of EtBr efflux (IC_50_ 140 µM)	[59]
Baicalein (**12**)	*Scutellaria baicalensis*	*S. aureus* OM481, OM584; *E. coli* KAM32/pTZ1252	TetK	Inhibition of tetracyclin efflux at 25 mg/L	[67]
	n.a.	*M. aurum* ATCC 23366	n.s.	Weak increase of EtBr accumulation	[68]
	n.a.	*M. smegmatis* mc^2^ 155	n.s.	Moderate increase of EtBr accumulation	[68]
Chrysoeriol (**13**)	*Artemisia rupestris*	*S. aureus* 1199B	NorA	Significant synergistic effects with norfloxacin; EtBr efflux inhibition; decreased the expression of NorA at mRNA level	[69]
	*Artemisia rupestris*	EMRSA-15, EMRSA-16	n.s.	Significant synergistic effect with ciprofloxacin and oxacillin; strong inhibitory effect on EtBr efflux in EMRSA-16	[69]
6-Desmethyl sideroxylin (**14**)	*Hydrastis canadensis*	*S. aureus* NCTC 8325-4, K1758	NorA	Inhibition of EtBr efflux in wild type strain, but not in *norA* knockout mutant	[70]
8-Desmethyl sideroxylin (**15**)	*Hydrastis canadensis*	*S. aureus* NCTC 8325-4, K1758	NorA	Inhibition of EtBr efflux in wild type strain, but not in *norA* knockout mutant	[70]
Diosmetin (**16**)	*Sophora moorcroftiana*	*S. aureus* 1199B	NorA	Synergy with norfloxacin, streptomycin and ciprofloxacin; no effect in EtBr efflux assay	[71]
	*Sophora moorcroftiana*	EMRSA-15	n.s.	Synergy with norfloxacin, streptomycin and cipro-floxacin	[71]
	n.a.	*S. aureus* RN4222	NorA	Increasing antibacterial activity of berberin	[72]
Luteolin (**17**)	n.a.	*M. smegmatis* mc^2^ 155	n.s.	Weak inhibition (ca. 20%) of EtBr efflux at 160 µM	[49]
	n.a.	*S. aureus* NCTC 8325-4	n.s.	Dose dependent inhibition of EtBr efflux (IC_50_ 260 µM)	[59]
Nobiletin (**18**)	n.a.	*M. aurum* ATCC 23366	n.s.	Strong increase of EtBr accumulation	[68]
	n.a.	*M. smegmatis* mc^2^ 155	n.s.	Moderate increase of EtBr accumulation	[68]
Skullcapflavone II (**19**)	*Scutellaria baicalensis*	*M. aurum* ATCC 23366	n.s.	Weak increase of EtBr accumulation	[68]
	*Scutellaria baicalensis*	*M. smegmatis* mc^2^ 155	n.s.	Strong increase of EtBr accumulation	[68]
Tangeretin (**20**)	n.a.	*M. aurum* ATCC 23366	n.s.	Moderate increase of EtBr accumulation	[68]
	n.a.	*M. smegmatis* mc^2^ 155	n.s.	Weak increase of EtBr accumulation	[68]
Wogonin (**21**)	n.a.	*M. aurum* ATCC 23366	n.s.	Weak increase of EtBr accumulation; no activity on *M. smegmatis* mc^2^ 155	[68]
*Flavonols*					
Chrysosplenetin (**22**)	*Artemisia rupestris*	*S. aureus* 1199B	NorA	Significant synergistic effects with norfloxacin; EtBr efflux inhibition; decreased expression of NorA at mRNA level	[69]
Galangin (**23**)	*Alpinia calcarata*	*S. aureus* 1199, 1199B, K1758	NorA	NorA inhibition (EtBr as substrate)	[65]
Kaempferol (**24**)	n.a.	*S. aureus* NCTC 8325-4	n.s.	Dose dependent inhibition of EtBr efflux (IC_50_ 66 µM)	[59]
	*Alpinia calcarata*	*S. aureus* 1199, 1199B, K1758	NorA	NorA inhibition (EtBr as substrate)	[65]
Kaempferol-3-*O*-α-L-(2,4-bis-*E*-p-coumaroyl)rhamnoside (**25**)	*Persea linguae*	*S. aureus* ATCC 29213, 1199B, K1758; everted membrane vesicles enriched with NorA	NorA	Strong inhibition (in combination with ciprofloxacin and EtBr); no effect in the NorA knockout strain K1758	[73]
Myricetin (**26**)	n.a.	*S. aureus* NCTC 8325-4	n.s.	Dose dependent inhibition of EtBr efflux (IC_50_ 240 µM)	[59]
Myricitrin (**27**)	n.a.	*S. aureus* 1199B	NorA	Decrease of norfloxacin and EtBr MIC	[62]
Penduletin (**28**)	*Artemisia rupestris*	*S. aureus* 1199B	NorA	Significant synergistic effects with norfloxacin; EtBr efflux inhibition; decreased the expression of NorA at mRNA level	[69]
Quercetin (**29**)	n.a.	*E. coli* BW25113 wild type and with *acrB* deletion	AcrAB-TolC	Inhibition of Nile red efflux at 200 µM; no synergy with tested antibiotics	[74]
	n.a.	*S. aureus* NCTC 8325-4	n.s.	Dose dependent inhibition of EtBr efflux (IC_50_ 250 µM)	[59]
Rhamnetin (**30**)	n.a.	*S. aureus* NCTC 8325-4	n.s.	Dose dependent inhibition of EtBr efflux (IC_50_ 60 µM)	[59]
Tiliroside (**31**)	*Herissantia tiubae*	*S. aureus* 1199B	NorA	8–16-fold reduction of norfloxacin MIC	[75]
*Flavonolignans*					
5´-Methoxyhydnocarpin D (**32**)	*Berberis sp.*	*S. aureus* RN4222, KLE 820	NorA	Increasing sensitivity to berberin	[37,76]
Silybin (**33**)	*Silybum marianum*	*S. aureus* RN4222, KLE 820	NorA	Weak increase of sensitivity to berberin	[76]
*Isoflavones*					
Biochanin A (**34**)	*Lupinus argenteus*	*S. aureus* NCTC 8325-4, KLE 8	NorA	Increased accumulation of berberin; decreasing MIC of berberin and norfloxacin	[51]
	n.a.	*M. smegmatis* mc^2^ 155	n.s.	Inhibition of EtBr efflux at 80 µM comparable to verapamil	[49]
Daidzein (**35**)	n.a.	*E. coli*	AcrB	Interaction with the distal binding pocket of AcrB (in- silico); increasing EtBr accumulation	[77]
	n.a.	*P. aeruginosa*	MexB	Interaction with the distal binding pocket of MexB (in-silico); increasing EtBr accumulation	[77]
Genistein (**36**)	*Lupinus argenteus*	*S. aureus* NCTC 8325-4, KLE-8	NorA	Increased accumulation of berberin; decreasing MIC of berberin and norfloxacin	[51]
	*Sophora moorcroftia-na*	*S. aureus* 1199B	NorA	Synergy with norfloxacin; marginal effect in EtBr efflux assay	[71]
Orobol (**37**)	*Lupinus argenteus*	*S. aureus* NCTC 8325-4, KLE-8	NorA	Increased accumulation of berberin; decreasing MIC of berberin and norfloxacin	[51]

n.a. not available; n.s. not specified.

## Data Availability

Not applicable.

## Sample Availability

Samples of the compounds are not available from the authors.

## References

[1] Martínez J.L., Rojo F. (2011). Metabolic regulation of antibiotic resistance. FEMS Microbiol. Rev..

[2] Poole K. (2012). Bacterial stress responses as determinants of antimicrobial resistance. J. Antimicrob. Chemother..

[3] Baharoglu Z., Mazel D. (2014). SOS, the formidable strategy of bacteria against aggressions. FEMS Microbiol. Rev..

[4] Alekshun M.N., Levy S.B. (2007). Molecular mechanisms of antibacterial multidrug resistance. Cell.

[5] Fernández L., Hancock R.E.W. (2012). Adaptive and mutational resistance: Role of porins and efflux pumps in drug resistance. Clin. Microbiol. Rev..

[6] Li X.-Z., Plésiat P., Nikaido H. (2015). The challenge of efflux-mediated antibiotic resistance in Gram-negative bacteria. Clin. Microbiol. Rev..

[7] van Bambeke F., Balzi E., Tulkens P.M. (2000). Antibiotic efflux pumps. Biochem. Pharmacol..

[8] Saier M.H., Paulsen I.T. (2001). Phylogeny of multidrug transporters. Semin. Cell Dev. Biol..

[9] Hassan K.A., Liu Q., Henderson P.J.F., Paulsen I.T. (2015). Homologs of the Acinetobacter baumannii AceI Transporter Represent a New Family of Bacterial Multidrug Efflux Systems. mBio.

[10] van Bambeke F., Glupczynski Y., Plésiat P., Pechère J.C., Tulkens P.M. (2003). Antibiotic efflux pumps in prokaryotic cells: Occurrence, impact on resistance and strategies for the future of antimicrobial therapy. J. Antimicrob. Chemother..

[11] Du D., Wang-Kan X., Neuberger A., van Veen H.W., Pos K.M., Piddock L.J.V., Luisi B.F. (2018). Multidrug efflux pumps: Structure, function and regulation. Nat. Rev. Microbiol..

[12] Schmalstieg A.M., Srivastava S., Belkaya S., Deshpande D., Meek C., Leff R., van Oers N.S.C., Gumbo T. (2012). The antibiotic resistance arrow of time: Efflux pump induction is a general first step in the evolution of mycobacterial drug resistance. Antimicrob. Agents Chemother..

[13] Nguyen F., Starosta A.L., Arenz S., Sohmen D., Dönhöfer A., Wilson D.N. (2014). Tetracycline antibiotics and resistance mechanisms. Biol. Chem..

[14] Fonseca J.D., Knight G.M., McHugh T.D. (2015). The complex evolution of antibiotic resistance in Mycobacterium tuberculosis. Int. J. Infect. Dis..

[15] Machado D., Couto I., Perdigão J., Rodrigues L., Portugal I., Baptista P., Veigas B., Amaral L., Viveiros M. (2012). Contribution of Efflux to the Emergence of Isoniazid and Multidrug Resistance in Mycobacterium tuberculosis. PLoS ONE.

[16] Rahman T., Yarnall B., Doyle D.A. (2017). Efflux drug transporters at the forefront of antimicrobial resistance. Eur. Biophys. J..

[17] Piddock L.J.V. (2006). Clinically relevant chromosomally encoded multidrug resistance efflux pumps in bacteria. Clin. Microbiol. Rev..

[18] Li X.-Z., Nikaido H. (2009). Efflux-mediated drug resistance in bacteria. Drugs.

[19] Hernando-Amado S., Blanco P., Alcalde-Rico M., Corona F., Reales-Calderón J.A., Sánchez M.B., Martínez J.L. (2016). Multidrug efflux pumps as main players in intrinsic and acquired resistance to antimicrobials. Drug Resist. Updat..

[20] Li X.-Z., Zhang L., Nikaido H. (2004). Efflux pump-mediated intrinsic drug resistance in Mycobacterium smegmatis. Antimicrob. Agents Chemother..

[21] Hernández A., Sánchez M.B., Martínez J.L. (2011). Quinolone Resistance: Much More than Predicted. Front. Microbio..

[22] Nikaido H., Pagès J.-M. (2012). Broad-specificity efflux pumps and their role in multidrug resistance of Gram-negative bacteria. FEMS Microbiol. Rev..

[23] Weston N., Sharma P., Ricci V., Piddock L.J. (2018). Regulation of the AcrAB-TolC efflux pump in Enterobacteriaceae. Res. Microbiol..

[24] Ramaswamy V.K., Cacciotto P., Malloci G., Ruggerone P., Vargiu A.V., Li X.-Z., Elkins C.A., Zgurskaya H.I. (2016). Multidrug Efflux Pumps and Their Inhibitors Characterized by Computational Modeling. Efflux-Mediated Antimicrobial Resistance in Bacteria: Mechanisms, Regulation and Clinical Implications.

[25] Pagès J.-M., Amaral L., Fanning S. (2011). An original deal for new molecule: Reversal of efflux pump activity, a rational strategy to combat gram-negative resistant bacteria. Curr. Med. Chem..

[26] Truong-Bolduc Q.C., Hooper D.C. (2010). Phosphorylation of MgrA and its effect on expression of the NorA and NorB efflux pumps of Staphylococcus aureus. J. Bacteriol..

[27] Tsao S., Weber S., Cameron C., Nehme D., Ahmadzadeh E., Raymond M. (2016). Positive regulation of the Candida albicans multidrug efflux pump Cdr1p function by phosphorylation of its N-terminal extension. J. Antimicrob. Chemother..

[28] Chan B.C.L., Ip M., Gong H., Lui S.L., See R.H., Jolivalt C., Fung K.P., Leung P.C., Reiner N.E., Lau C.B.S. (2013). Synergistic effects of diosmetin with erythromycin against ABC transporter over-expressed methicillin-resistant Staphylococcus aureus (MRSA) RN4220/pUL5054 and inhibition of MRSA pyruvate kinase. Phytomedicine.

[29] van Bambeke F., Pages J.-M., Lee V.J., Atta-ur-Rahman, Iqbal Choudhary M. (2012). Inhibitors of Bacterial Efflux Pumps as Adjuvants in Antibacterial Therapy and Diagnostic Tools for Detection of Resistance by E. Frontiers in Anti-Infective Drug Discovery.

[30] Bohnert J.A., Kern W.V., Li X.-Z., Elkins C.A., Zgurskaya H.I. (2016). Antimicrobial Drug Efflux Pump Inhibitors. Efflux-Mediated Antimicrobial Resistance in Bacteria: Mechanisms, Regulation and Clinical Implications.

[31] Abreu A.C., Mcbain A.J., Simões M. (2012). Plants as sources of new antimicrobials and resistance-modifying agents. Nat. Prod. Rep..

[32] Tegos G.P., Haynes M., Strouse J.J., Khan M.M.T., Bologa C.G., Oprea T.I., Sklar L.A. (2011). Microbial efflux pump inhibition: Tactics and strategies. Curr. Pharm. Des..

[33] Prasch S., Bucar F. (2015). Plant derived inhibitors of bacterial efflux pumps: An update. Phytochem. Rev..

[34] Martinez J.L., Sánchez M.B., Martínez-Solano L., Hernandez A., Garmendia L., Fajardo A., Alvarez-Ortega C. (2009). Functional role of bacterial multidrug efflux pumps in microbial natural ecosystems. FEMS Microbiol. Rev..

[35] Willers C., Wentzel J.F., Du Plessis L.H., Gouws C., Hamman J.H. (2017). Efflux as a mechanism of antimicrobial drug resistance in clinical relevant microorganisms: The role of efflux inhibitors. Expert Opin. Ther. Targets.

[36] Stavri M., Piddock L.J.V., Gibbons S. (2007). Bacterial efflux pump inhibitors from natural sources. J. Antimicrob. Chemother..

[37] Stermitz F.R., Lorenz P., Tawara J.N., Zenewicz L.A., Lewis K. (2000). Synergy in a medicinal plant: Antimicrobial action of berberine potentiated by 5′-methoxyhydnocarpin, a multidrug pump inhibitor. PNAS.

[38] Mikulášová M., Chovanová R., Vaverková Š. (2016). Synergism between antibiotics and plant extracts or essential oils with efflux pump inhibitory activity in coping with multidrug-resistant staphylococci. Phytochem. Rev..

[39] Romiti N., Pellati F., Nieri P., Benvenuti S., Adinolfi B., Chieli E. (2008). P-Glycoprotein inhibitory activity of lipophilic constituents of Echinacea pallida roots in a human proximal tubular cell line. Planta Med..

[40] Chieli E., Romiti N., Catiana Zampini I., Garrido G., Inés Isla M. (2012). Effects of Zuccagnia punctata extracts and their flavonoids on the function and expression of ABCB1/P-glycoprotein multidrug transporter. J. Ethnopharmacol..

[41] Andersen O.M., Markham K.R. (2005). Flavonoids.

[42] Bucar F., Xiao J., Ochensberger S., Xiao J., Sarker S.D., Asakawa Y. (2021). Flavonoid C-Glycosides in Diets. Handbook of Dietary Phytochemicals.

[43] Ferreira A., Pousinho S., Fortuna A., Falcão A., Alves G. (2015). Flavonoid compounds as reversal agents of the P-glycoprotein-mediated multidrug resistance: Biology, chemistry and pharmacology. Phytochem. Rev..

[44] Singh P., Anand A., Kumar V. (2014). Recent developments in biological activities of chalcones: A mini review. Eur. J. Med. Chem..

[45] Parveen Z., Brunhofer G., Jabeen I., Erker T., Chiba P., Ecker G.F. (2014). Synthesis, biological evaluation and 3D-QSAR studies of new chalcone derivatives as inhibitors of human P-glycoprotein. Bioorg. Med. Chem..

[46] Blair J.M.A., Piddock L.J.V. (2016). How to Measure Export via Bacterial Multidrug Resistance Efflux Pumps. mBio.

[47] Wang Y., Venter H., Ma S. (2016). Efflux Pump Inhibitors: A Novel Approach to Combat Efflux-Mediated Drug Resistance in Bacteria. Curr. Drug Targets.

[48] Spengler G., Kincses A., Gajdács M., Amaral L. (2017). New Roads Leading to Old Destinations: Efflux Pumps as Targets to Reverse Multidrug Resistance in Bacteria. Molecules.

[49] Lechner D., Gibbons S., Bucar F. (2008). Plant phenolic compounds as ethidium bromide efflux inhibitors in Mycobacterium smegmatis. J. Antimicrob. Chemother..

[50] Gröblacher B., Kunert O., Bucar F. (2012). Compounds of Alpinia katsumadai as potential efflux inhibitors in Mycobacterium smegmatis. Bioorg. Med. Chem..

[51] Morel C., Stermitz F.R., Tegos G., Lewis K. (2003). Isoflavones as potentiators of antibacterial activity. J. Agric. Food Chem..

[52] Kurinčič M., Klančnik A., Smole Možina S. (2012). Effects of efflux pump inhibitors on erythromycin, ciprofloxacin, and tetracycline resistance in Campylobacter spp. isolates. Microb. Drug Resist..

[53] Gibbons S., Moser E., Kaatz G.W. (2004). Catechin gallates inhibit multidrug resistance (MDR) in Staphylococcus aureus. Planta Med..

[54] Viveiros M., Martins A., Paixão L., Rodrigues L., Martins M., Couto I., Fähnrich E., Kern W.V., Amaral L. (2008). Demonstration of intrinsic efflux activity of Escherichia coli K-12 AG100 by an automated ethidium bromide method. Int. J. Antimicrob. Agents.

[55] Rodrigues L., Ramos J., Couto I., Amaral L., Viveiros M. (2011). Ethidium bromide transport across Mycobacterium smegmatiscell-wall: Correlation with antibiotic resistance. BMC Microbiol..

[56] Rodrigues L., Aínsa J.A., Viveiros M., Parish T., Kumar A. (2021). Measuring Efflux and PermeabilityPermeability in Mycobacteria. Mycobacteria Protocols.

[57] Araya-Cloutier C., Vincken J.P., van de Schans M.G., Hageman J., Schaftenaar G., den Besten H.M., Gruppen H. (2018). QSAR-based molecular signatures of prenylated (iso)flavonoids underlying antimicrobial potency against and membrane-disruption in Gram positive and Gram negative bacteria. Sci. Rep..

[58] Kovač J., Šimunović K., Wu Z., Klančnik A., Bucar F., Zhang Q., Možina S.S. (2015). Antibiotic resistance modulation and modes of action of (-)-α-Pinene in Campylobacter jejuni. PLoS ONE.

[59] Brown A.R., Ettefagh K.A., Todd D., Cole P.S., Egan J.M., Foil D.H., Graf T.N., Schindler B.D., Kaatz G.W., Cech N.B. (2015). A mass spectrometry-based assay for improved quantitative measurements of efflux pump inhibition. PLoS ONE.

[60] Brown A.R., Ettefagh K.A., Todd D.A., Cole P.S., Egan J.M., Foil D.H., Lacey E.P., Cech N.B. (2021). Bacterial efflux inhibitors are widely distributed in land plants. J. Ethnopharmacol..

[61] Belofsky G., Percivill D., Lewis K., Tegos G.P., Ekart J. (2004). Phenolic metabolites of Dalea versicolor that enhance antibiotic activity against model pathogenic bacteria. J. Nat. Prod..

[62] Diniz-Silva H.T., Magnani M., de Siqueira S., de Souza E.L., de Siqueira-Júnior J.P. (2017). Fruit flavonoids as modulators of norfloxacin resistance in Staphylococcus aureus that overexpresses norA. LWT-Food Sci. Technol..

[63] Kurinčič M., Klančnik A., Smole Možina S. (2012). Epigallocatechin gallate as a modulator of Campylobacter resistance to macrolide antibiotics. Int. J. Antimicrob. Agents.

[64] Saiful A.J., Ling S.K., Mastura M., Mazura M.I., Salbiah M., Shuhaimi M., Abdul M.A. (2012). Efflux inhibitory activity of flavonoids from Chromolaena odorata against selected methicillin-resistant Staphylococcus aureus (MRSA) isolates. Afr. J. Microbiol. Res..

[65] Randhawa H.K., Hundal K.K., Ahirrao P.N., Jachak S.M., Nandanwar H.S. (2016). Efflux pump inhibitory activity of flavonoids isolated from Alpinia calcarata against methicillin-resistant Staphylococcus aureus. Biologia.

[66] Sun Z.-L., Sun S.-C., He J.-M., Lan J.-E., Gibbons S., Mu Q. (2020). Synergism of sophoraflavanone G with norfloxacin against effluxing antibiotic-resistant Staphylococcus aureus. Int. J. Antimicrob. Agents.

[67] Fujita M., Shiota S., Kuroda T., Hatano T., Yoshida T., Mizushima T., Tsuchiya T. (2005). Remarkable Synergies between Baicalein and Tetracycline, and Baicalein and β-Lactams against Methicillin-Resistant Staphylococcus aureus. Microbiol. Immunol..

[68] Solnier J., Bucar F., Martin L., Bhakta S. (2020). Flavonoids as Novel Efflux Pump Inhibitors and Antimicrobials Against Both Environmental and Pathogenic Intracellular Mycobacterial Species. Molecules.

[69] Lan J.-E., Li X.-J., Zhu X.-F., Sun Z.-L., He J.-M., Zloh M., Gibbons S., Mu Q. (2021). Flavonoids from Artemisia rupestris and their synergistic antibacterial effects on drug-resistant Staphylococcus aureus. Nat. Prod. Res..

[70] Junio H.A., Sy-Cordero A.A., Ettefagh K.A., Burns J.T., Micko K.T., Graf T.N., Richter S.J., Cannon R.E., Oberlies N.H., Cech N.B. (2011). Synergy-directed fractionation of botanical medicines: A case study with goldenseal (Hydrastis canadensis). J. Nat. Prod..

[71] Wang S.-Y., Sun Z.-L., Liu T., Gibbons S., Zhang W.-J., Qing M. (2014). Flavonoids from Sophora moorcroftiana and their Synergistic Antibacterial Effects on MRSA. Phytother. Res..

[72] Guz N.R., Stermitz F.R., Johnson J.B., Beeson T.D., Willen S., Hsiang J.-F., Lewis K. (2001). Flavonolignan and Flavone Inhibitors of a Staphylococcus aureus Multidrug Resistance Pump: Structure−Activity Relationships. J. Med. Chem..

[73] Holler J.G., Christensen S.B., Slotved H.-C., Rasmussen H.B., Gúzman A., Olsen C.-E., Petersen B., Mølgaard P. (2012). Novel inhibitory activity of the Staphylococcus aureus NorA efflux pump by a kaempferol rhamnoside isolated from Persea lingue Nees. J. Antimicrob. Chemother..

[74] Ohene-Agyei T., Mowla R., Rahman T., Venter H. (2014). Phytochemicals increase the antibacterial activity of antibiotics by acting on a drug efflux pump. MicrobiologyOpen.

[75] Falcão-Silva V.S., Silva D.A., Souza M.d.F.V., Siqueira-Junior J.P. (2009). Modulation of drug resistance in Staphylococcus aureus by a kaempferol glycoside from Herissantia tiubae (Malvaceae). Phytother. Res..

[76] Stermitz F.R., Tawara-Matsuda J., Lorenz P., Mueller P., Zenewicz L., Lewis K. (2000). 5’-Methoxyhydnocarpin-D and pheophorbide A: Berberis species components that potentiate berberine growth inhibition of resistant Staphylococcus aureus. J. Nat. Prod..

[77] Aparna V., Dineshkumar K., Mohanalakshmi N., Velmurugan D., Hopper W. (2014). Identification of Natural Compound Inhibitors for Multidrug Efflux Pumps of Escherichia coli and Pseudomonas aeruginosa Using In Silico High-Throughput Virtual Screening and In Vitro Validation. PLoS ONE.

[78] Hellewell L., Bhakta S. (2020). Chalcones, stilbenes and ketones have anti-infective properties via inhibition of bacterial drug-efflux and consequential synergism with antimicrobial agents. Access Microbiol..

[79] Cannalire R., Machado D., Felicetti T., Santos Costa S., Massari S., Manfroni G., Barreca M.L., Tabarrini O., Couto I., Viveiros M. (2017). Natural isoflavone biochanin A as a template for the design of new and potent 3-phenylquinolone efflux inhibitors against Mycobacterium avium. Eur. J. Med. Chem..

[80] Chambers C.S., Viktorova J., Rehorova K., Biedermann D., Turkova L., Macek T., Kren V., Valentova K. (2020). Defying Multidrug Resistance! Modulation of Related Transporters by Flavonoids and Flavonolignans. J. Agric. Food Chem..

[81] Fang Y., Liang F., Xia M., Cao W., Pan S., Wu T., Xu X. (2021). Structure-activity relationship and mechanism of flavonoids on the inhibitory activity of P-glycoprotein (P-gp)-mediated transport of rhodamine123 and daunorubicin in P-gp overexpressed human mouth epidermal carcinoma (KB/MDR) cells. Food Chem. Toxicol..

[82] Marques S.M., Šupolíková L., Molčanová L., Šmejkal K., Bednar D., Slaninová I. (2021). Screening of Natural Compounds as P-Glycoprotein Inhibitors against Multidrug Resistance. Biomedicines.

